# Subjective Age and Job Satisfaction: A Moderated Mediation Model of Job Burnout and Chronological Age

**DOI:** 10.3389/fpubh.2020.00062

**Published:** 2020-03-06

**Authors:** Muhammad Khalid Anser, Moazzam Ali, Farooq Anwar, Muhammad Usman

**Affiliations:** ^1^School of Public Administration, Xi'an University of Architecture and Technology, Xi'an, China; ^2^Department of Management Sciences, University of Okara, Okara, Pakistan; ^3^Lahore Business School, The University of Lahore, Lahore, Pakistan; ^4^Department of Management Sciences, COMSATS University Islamabad, Lahore, Pakistan

**Keywords:** subjective age, chronological age, job satisfaction, job burnout, moderated-mediation

## Abstract

Corresponding to the growing calls for theory-driven research on the age-job satisfaction association, the present study investigated direct and indirect (via job burnout) relationships between subjective age (felt age) and job satisfaction. The study also examined the moderating role of chronological age on both direct and indirect (via job burnout) relationships between subjective age and job satisfaction. Survey data were collected in three waves (2 months apart) from 355 employees in 62 firms operating in various service and manufacturing industry sectors in Pakistan. Data were analyzed using structural equation modeling, PROCESS macro for SPSS, and bootstrapping technique. The results showed subjective age was negatively related to job satisfaction, both directly (β = −0.19, *p* < 0.001) and indirectly, via job burnout (β = −0.09, bootstrap 95% confidence interval limits did not overlap with zero; lower limit = −0.15, upper limit = −0.04). Interestingly, the interaction term (relative subjective age × chronological age) had a significant negative effect on the direct negative association between subjective age and job satisfaction (*B* = −0.12, *p* < 0.05) and a significant positive effect on the direct positive relationship between subjective age and job burnout (*B* = 0.14, *p* < 0.01), showing that chronological age moderated the direct relationships of subjective age with job satisfaction and job burnout, respectively. Importantly, the results showed that chronological age moderated the indirect association (via job burnout) between subjective age and job satisfaction [bootstrap estimate = −0.025, bias-corrected confidence interval (−0.06, −0.002)]. The present study contributed to the literature on the age-job satisfaction association by suggesting subjective age as an alternative vantage point to look at this link between age and job satisfaction. The findings carry useful practical implications that can help managers counter age stereotyping, employees' feelings of job burnout, and a low level of employees' job satisfaction.

## Introduction

Employee job satisfaction has received much attention from researchers and practitioners because of its constructive influence on employees' productivity and performance (1, 2). A plethora of studies have revealed that job satisfaction reduces absenteeism, employee turnover, and counterproductive behaviors (1, 3) and enhances employees' motivation, trust, and citizenship behaviors (4, 5). Therefore, the focus of management on job satisfaction is a vital aspect of managing human resources and employees' behaviors and performance outcomes (6, 7).

Age is one of the widely researched predictors of job satisfaction (8–12). Despite valuable contributions to both theory and practice, the literature on the age and job satisfaction relationship contains several ambiguities and unaddressed questions. A key concern relevant to our study is the way prior studies have treated age in the age-job satisfaction relationship. Past research has made the simplest choice of the age—chronological age (actual age in years)—to showcase different types of relationships with job satisfaction, such as linear positive and negative, U-shaped, and curvilinear relationships (3, 4, 9, 11). We argue that the chronological age offers an important but a restrictive view of the age-job satisfaction relationship. People with the same chronological age can have different subjective age—subjective views about their age (13–15). The assumption that individuals sharing the same chronological age groups will exhibit similar attitudes and behaviors is flawed (13, 15, 16). Rather, people with the same age can behave and act differently depending on their subjective age, which can be equal to, and lower or higher than, their chronological age (13, 17, 18). Indeed, several studies have revealed that the explanatory power of subjective age is stronger than the chronological age [e.g., (13, 15, 16, 19–21)]. Moreover, there are growing calls for theory-driven research on the association of age with employees' work-related outcomes (22, 23). Thus, we understand that subjective age at work (subjective age henceforth) may have greater relevance in explaining the variations in employees' attitudes, behaviors, and performance outcomes (22, 23) and can have important implications for managers concerned about employees' work-related outcomes. However, despite important theoretical and practical implications, as noted in previous studies (15, 24) there is a surprising scarcity of studies on the subjective age-job satisfaction relationship.

To fill in these research gaps, we draw on the socioemotional selectivity theory (SST) (25) and the conservation of resources (COR) theory (26, 27) to theorize a negative association of subjective age with job satisfaction. Furthermore, given the scarcity of studies on the subjective age-job satisfaction connection, little is known about the mechanisms underlying the subjective age-job satisfaction link, leaving it unknown why subjective age is negatively related to job satisfaction. To address this research void, we propose that job burnout—an employee's perception that his/her personal resources, such as physical and emotional energies are gradually diminishing (28)—mediates the subjective age-job satisfaction association. We focus on job burnout, as it carries a significant cost for organizations because of its destructive effects on employees' work-related outcomes (29, 30). Job burnout deteriorates employees' mental, physical, and behavioral health (31, 32). Job burnout also results in reduced job satisfaction, turnover, increased absenteeism, and lowered commitment (29, 33).

**Figure 1:**
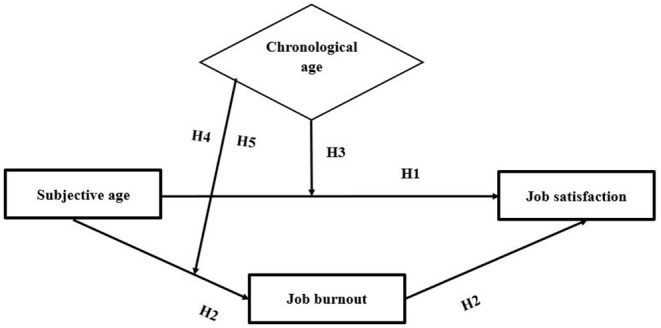
The proposed model.

Finally, previous studies (16, 34, 35) suggest that the consequences of subjective age vary across different age groups. For instance, Fang and Galambos (35) and Bergland et al. (34) suggest that older subjective age bias among middle-aged and old-aged individuals is indicative of low levels of mental and physical energy and life satisfaction, while the older subjective age bias among young people might reflect their maturity, a sense of inclusion, and life satisfaction (16, 24, 34). Drawing on these arguments, we argue that the relationships of subjective age with job burnout and job satisfaction are not straightforward; rather, these relationships may be contingent on employees' chronological age. However, to date, not a single study has hypothesized and empirically shown that chronological age acts as a boundary condition of the subjective age-job satisfaction association and the subjective age-job burnout linkage. The present study addresses these gaps by proposing and revealing that chronological age moderates both direct and indirect subjective age-job satisfaction associations and the subjective age-job burnout association. The proposed model is presented in Figure 1.

## Hypotheses Development

### Subjective Age and Job Satisfaction

Job satisfaction refers to an employee's positive emotions and attitude toward the job, work environment, and his/her role in the organization (36). Scholars have shown a consensus about the imperative role of job satisfaction in the improvement of organizational practices and performance (37–39). Moreover, job satisfaction is one of the significant predictors of employees' vocational happiness, commitment, improved productivity, and citizenship behaviors (7, 38).

The SST posits that in younger years, people often perceive an open-ended future time perspective (FTP), while in older years, they follow a limited FTP (40, 41). In a work context, younger employees prefer to pursue goals that open future career growth opportunities by focusing on the acquisition of new knowledge and skills. In contrast, when employees' chronological age increases, they often demonstrate a low level of interest in professional training and development, the acquisition of new skills, and long-term success (25, 42). Therefore, drawing upon the SST, we argue that employees who report their subjective age higher than their chronological age, they follow a limited FTP (40, 41) and thus, can demonstrate a low level of interest in work activities, such as learning and development, that are linked with the long-term success. In other words, based on the SST, we understand that employees who report subjective age higher than their chronological age may lack important resources, such as passion, energy, and skills.

Furthermore, the COR theory (26) posits that employees endeavor to gain, retain and protect valued resources, such as knowledge, skills, conditions, objects, social support, and energy. The COR theory further suggests that losing or anticipating a loss of valued resources results in burnout at work, a discrepancy between expected and actual performance, and a low level of job satisfaction. Moreover, initial resource loss results in future resource loss. As employees try to invest resources from one domain to the other domains, in which the loss has occurred or the loss is anticipated, the loss or anticipation of the loss in one domain depletes resources in other domains. Thus, building on the COR theory (26), we suggest that employees with older subjective age bias may be deficient in resources (e.g., such as passion, energy, and skills) required to perform their work roles that can lead to a discrepancy between expected and actual performance and a low level of job satisfaction.

Additionally, empirical studies show that youthful subjective age is positively associated with employees' self-efficacy, productivity, performance, and work motivation (23, 43), suggesting that youthful subjective age can be positively related to job satisfaction. Moreover, although empirical studies on the subjective age-satisfaction link in the work context are non-existing, a study in a non-work context (44) showed that youthful subjective age bias is positively related to life satisfaction. Thus, the following hypothesis has been developed.

***Hypothesis 1***. *Subjective age is negatively related to employee job satisfaction*.

### Job Burnout as a Mediator

To establish job burnout as a mediator of the subjective age-job satisfaction association, we build on the SST (25) and the COR theory (26). The SST suggests that during the lifespan, individuals' time horizon shrinks because of the shifts in their FTP—from an unlimited, an open-ended FTP to a limited FTP—and consequently, they demonstrate a low level of interest passion and energy for the work, professional development, and the acquisition of new skills (42). That is, due to the limited FTP, employees with older subjective age bias can face a shortage of skills and knowledge, given their reduced focus on skill development (42) that, as posited by the COR theory, can deplete their mental and physical energies.

Additionally, the literature on the links between subjective age and health shows that older subjective age bias negatively is negatively related to individuals' health and well-being (45, 46). Thus, the literature suggests that subjective age higher than the chronological age is indicative of poor physical and mental energy (47, 48). Therefore, we infer that middle-aged employees with older subjective age bias (subjective age higher than their chronological age) feel low levels of energy and demonstrate a reduced level of interest in their work roles that if seen through the lens of the COR theory, constitute a loss of important personal resources at work. According to the COR theory, the continual depletion of such resources as physical and emotional energy and professional knowledge can result in job burnout, which is negatively related to several employees' constructive outcomes, including job satisfaction (27). Thus, based on the COR theory, it can be argued that that continual resources' depletion (e.g., a loss of physical and mental energy) or the absence of resources leads to job burnout (26). Job burnout, in turn, negatively affects various work-related outcomes, such as job satisfaction (49, 50). In sum, we understand that job burnout can explain why subjective age is negatively related to job satisfaction.

***Hypothesis 2***. *Job burnout mediates the negative relationship between subjective age and job satisfaction*.

### Chronological Age as a Moderator

Past research has revealed that perception of subjective age can vary across individuals within the same chronological age group, and that can result in their different perceptions of mental and physical health, subjective well-being, and life satisfaction (15, 16). For instance, middle-aged and older adults with youthful subjective age perceptions reported better physical and emotional health than those with older subjective age bias (34, 51). Indeed, youthful subjective age bias among middle-aged and older adults has been found to have positive associations with mental and physical health, subjective well-being, a sense of inclusion, and life satisfaction (34, 52). Likewise, younger individuals with the same chronological age can have different perceptions of their subjective age and report different perceptions about health and well-being and life satisfaction (15).

Furthermore, the literature on subjective age suggests that older subjective age bias among younger people reflects maturity and confidence that help them gain success and life satisfaction (24, 35, 53). On the contrary, older subjective age bias among middle-aged or older adults is indicative of poor mental and physical health and low levels of energy, passion, and life satisfaction (34). Seen through the lens of the COR theory, poor mental and physical energies among middle-aged employees can negatively affect their work-related attitudes, such as job satisfaction, as well as trigger the feelings of job burnout. Drawing from the above discussion, we understand that as compared to older subjective age bias among younger employees, older subjective age bias among middle-aged employees can have a more profound effect on job burnout and job satisfaction. Thus, the present study postulates the following hypotheses. It is worth noting that following Bellingtier and Neupert (24) and Shinan-Altman and Werner (15), the present study devised a criterion to divide the chronological age of the respondents into two groups—young employees and middle-aged employees.

***Hypothesis 3***. *Employees' chronological age moderates the negative relationship between subjective age and job satisfaction, such that the relationship is stronger for middle-aged employees than for younger employees*.

***Hypothesis 4***. *Employees' chronological age moderates the positive relationship between subjective age and job burnout, such that the relationship is stronger for middle-aged employees than for younger employees*.

Moreover, as we have alluded above (hypothesis 4), the positive subjective age-job burnout link is stronger for middle-aged employees than for younger employees, suggesting that the mediating effect of job burnout on the negative subjective age-job satisfaction association is contingent on employees' chronological age. Therefore, from a statistical perspective, this study presents a case of moderated mediation (54). More specifically, the present work predicts that the indirect negative relationship (via job burnout) between subjective age and job satisfaction can be stronger for middle-aged employees than for younger employees. Thus, the following hypothesis was developed.

***Hypothesis 5***. *Employees' chronological age moderates the indirect negative relationship (via job burnout) between subjective age and job satisfaction, such that the relationship is stronger for middle-aged employees than for younger employees*.

## Methods

### Data Collection and Analysis

We collected survey data from 355 employees in 62 Pakistani firms belonging to various service and manufacturing sectors. Employees' perceptions of subjective age at work can be different across industries, organizations, and professions. Therefore, following Hirschi (55) and Kreiner (56), we collected data from a heterogeneous sample to capture maximum variance in subjective age. Initially, 70 firms were randomly selected from the total firms listed on the Pakistan Stock Exchange. We managed access to 62 firms through personal and professional references. We collected data in three rounds separated by a 2 months lag. In the first round, data about work experience, education, chronological age, subjective age, and gender were collected. In the second round, we collected data about job burnout. Finally, in the third round, we collected data about job satisfaction. The survey questionnaire was translated into Urdu (the national language of Pakistan) using the Brislin's (57) back-translation method. The translated version of the questionnaire was pilot-tested with six academicians and 20 potential respondents.

A total of 600 employees were randomly selected from the employees of those companies that offered us access. Of these 600 randomly chosen employees, we were able to contact 576 employees, who were provided an information sheet containing information about the purpose of our research and the confidentiality promise. We received written consent for participation in data collection from 552 employees. In the first round, 390 respondents returned the completed questionnaires, while 372 and 368 employees returned the completed survey in the second and third rounds, respectively. After screening the data for negligence, we received 355 matched (59.16% net response rate) useable responses. The responses from the three rounds of data collection were matched using a unique code. The unique code was developed by asking the respondents to write the last two letters of their first name, the last two digits of their city code, the month of their birth, the last two digits of their mobile phone number, and the last two letters of their organization's name. These letters and digits were combined to generate the code. This information was asked in all the data collection rounds. In terms of gender, the sample included 52.4% males and 47.6% females. Respondents' average chronological age was 43 years. As far as education is concerned, 32.3% of the respondents had completed 12 years of schooling, 48% had undergraduate degrees, and 19.7% held master's degrees. We analyzed data using structural equation modeling (SEM) in AMOS 24.0, bootstrapping method, and [51] PROCESS macro for SPSS.

Data were collected in 2018. To minimize the common method bias, we followed Podsakoff et al. (58) recommendation and used a time-lagged design. A 2-months lag has been used in a number of prior publications (59–61). Moreover, we also used Harman's single-factor test (62) to diagnose the common method bias. For this purpose, we constrained all the items of the two latent constructs—job satisfaction and job burnout—into one factor, which resulted in explaining 47.64% of the total variance. Since the variance explained by a single factor did not exceed the cut-off value (50%), the common method was not a problem (62).

### Variables and Measures

#### Chronological and Subjective Age

To measure chronological age, the respondents were asked to write their actual age measured in years such as 25, 26, and 27 years. To operationalize subjective age, we followed (23, 47) and used the relative subjective age as a measure of subjective age. For this purpose, we asked employees how old they feel at work independent of his/her actual (chronological) age and then subtracted the chronological age from the subjective age (23). A positive value indicated that a respondent felt older compared to his/her chronological age, while a negative value indicated that a respondent felt younger compared to his/her chronological age. Moreover, employees with chronological age ≤44 years were considered as young, while employees with age >44 years were categorized as middle-aged. As the retirement age in Pakistan is 60 years, the present study did not take into the older age (age > 60 years) individuals.

#### Job Satisfaction

To measure job satisfaction, Macdonald and Maclntyre's (63) ten-item scale was used. A five-point Likert scale anchored on 1 (strongly disagree) to 5 (strongly agree) was used to gauge the responses. Sample items were “*I receive recognition for a job well-done”* and “*I feel close to the people at work.”*

#### Job Burnout

To measure job burnout, we used a seven-item scale (the Copenhagen burnout inventory to measure work-related burnout) developed and validated by Kristensen et al. (28). “*Do you feel worn out at the end of the day”* was a sample item. The responses were measured on a five-point Likert scale, anchored on 1(never) to 5 (always).

### Control Variables

Work experience and gender can affect job burnout (64, 65) and job satisfaction (8, 12) and thus, can confound the results. Moreover, employees' perceptions of job burnout and job satisfaction can vary across industries. Therefore, in the present study, we controlled for gender and industry type.

## Results

### Non-independence of the Data

As 355 respondents belonged to 62 organizations, we calculated the values of the intraclass correlation coefficient (ICC) for the outcome variable (job satisfaction) and the mediator (job burnout). ICC1 = 0.01 (ns) for job satisfaction and ICC1 = 0.00 (ns) for job burnout suggested that non-independence was not a problem in our data (66).

### Means and Correlations

The means, standard deviations, and inter-construct correlations are presented in Table 1. It is important to note that the mean value of relative subjective age was 2.96 years, suggesting that, on average, the respondents felt that their subjective age was higher than their chronological age.

**Table 1:** Means and correlations.

**Construct**	**Means**	**SD**	**1**	**2**	**3**	**4**	**5**	**6**
1. Relative subjective age	2.96	2.14						
2. Job burnout	3.46	0.99	0.35**					
3. Job satisfaction	2.50	0.90	−0.18**	−0.25**				
4. Age	43.10	7.66	0.26**	−0.04	0.03			
5. Gender	1.48	0.50	0.09	−0.02	−0.07	0.07		
6. Work experience	10.89	5.85	0.19*	0.01	0.02	0.76**	0.03	
7. Industry type	7.84	4.46	0.01	−0.05	0.01	0.03	0.01	0.12*

n, 355; *P < 0.05 level and **P < 0.01 level (2-tailed). SD, Standard deviation; Age, Chronological age.

### Measurement Model

Our measurement model was assessed using a confirmatory factor analysis (CFA). This model included job satisfaction, job burnout, and relative subjective age. The results demonstrated that the model had a good fit with the data, as chi-square (χ2) = 384.77, degrees of freedom (df) = 133, χ2/df = 2.89 <3, root mean square error of approximation (RMSEA) = 0.07 <0.08, incremental fit index (IFI) = Tucker-Lewis index (TLI) = comparative fit index (CFI) = 0.96 > 0.90 (67).

The values of Cronbach alpha (Table 2) for both the variables > 0.70 show that the level of internal consistency for both the variables (job satisfaction and job burnout) was satisfactory. The average variance extracted >0.50 suggested a satisfactory level of convergent validity (67). To assess the discriminant validity, we used the Fornel-Lacker criterion. It is evident from Table 2 that the square roots of average variance extracted of both reflective constructs—job burnout and job satisfaction—were greater than their corresponding inter-construct correlations. It is also evident from Table 2 that average shared variance < maximum shared variance < average variance extracted. Therefore, the measurement scales of job burnout and job satisfaction achieved satisfactory levels of discriminant validity.

**Table 2:** Discriminant validity, convergent validity, and internal consistency.

**Construct**	**1**	**2**	**α**	**AVE**	**MSV**	**ASV**
1. Job burnout	**0.90**		0.93	0.68	0.12	0.10
2. Job satisfaction	−0.28	**0.85**	0.95	0.70	0.08	0.05
3. Relative subjective age	0.35	−0.18	–	–	0.12	0.08

*AVE, Average variance extracted; MSV, Maximum shared variance; ASV, Average shared variance; α, Cronbach alpha. Bolded values on the diagonals (columns 2-3) are square root values of AVE*.

### Evaluation of the Structural Model and Mediation Results

First, we tested the direct relationship between relative subjective age and job satisfaction in the structural model (1). Direct (without mediator) negative association between relative subjective age and job satisfaction was found to be significant (β = −0.19, *p* < 0.001). The model (1) demonstrated a good with the data, as χ2 = 188.69, df = 70, χ2/df = 2.70 < 3, RMSEA = 0.07 < 0.08, and CFI = TLI = IFI = 0.97 > 0.90 (67). Thus, the results supported hypothesis 1.

**Figure 2:**
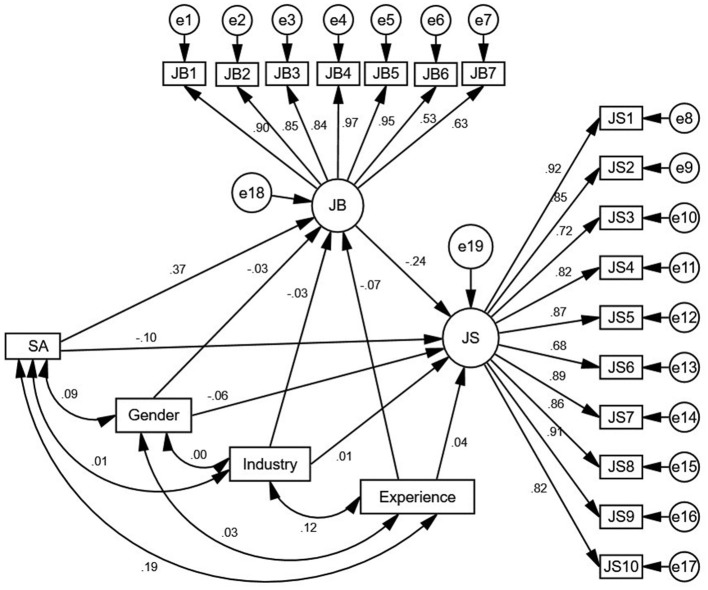
Structural model (2): Relationship between relative subjective age and job satisfaction via job burnout.

Then in the structural model (2), the mediator, job burnout was included in the relative subjective age-job satisfaction association. This model (2) also demonstrated a good fit with the data – χ2 = 432.10, df = 178, χ2/df = 2.43 < 3, RMSEA = 0.07 < 0.08, and CFI = TLI = IFI = 0.96 > 0.90 (67). The results of the structural model (2) suggested that job burnout can mediate the relative subjective age-job satisfaction relationship. The structural model (2) is depicted in Figure 2.

Finally, the significance of the mediating role of job burnout was tested using bootstrapping in AMOS 24.0 (a sample of size 2,000 was specified at a 95% confidence interval). Table 3 depicts the results.

**Table 3:** Direct and indirect effects and 95% confidence intervals for the model (2).

**Parameter**	**β**	**LL**	**UL**
**Standardized direct effects**			
Relative subjective age ➔Job burnout	0.37^*^	0.26	0.47
Relative subjective age ➔Job satisfaction	−0.12	−0.22	0.01
Job burnout ➔Job satisfaction	−0.24^*^	−0.36	−0.10
**Standardized indirect effects**			
Relative subjective age ➔Job burnout ➔Job satisfaction	−0.09^*^	−0.15	−0.04

* *Empirical 95% confidence interval does not overlap with zero. LL, Lower limit; UL, Upper limit*.

It is evident from Table 3 that after including job burnout as the mediator, the direct negative association of relative subjective age with job satisfaction became insignificant (β = −0.12, bootstrap 95% confidence interval limits overlapped with zero; lower limit = −0.22, upper limit = 0.01), while the relative subjective age-job satisfaction relationship via job burnout was significant (β = −0.09, β = −0.09, bootstrap 95% confidence interval limits did not overlap with zero; lower limit = −0.15, upper limit = −0.04). Moreover, as evident from Figure 2, the effect of control—gender, work experience, and industry type—on both job burnout and job satisfaction was insignificant. Thus, job burnout fully mediated the negative association between relative subjective age and job satisfaction. Thus, the results supported hypothesis 2.

### Moderation Results

**Figure 3:**
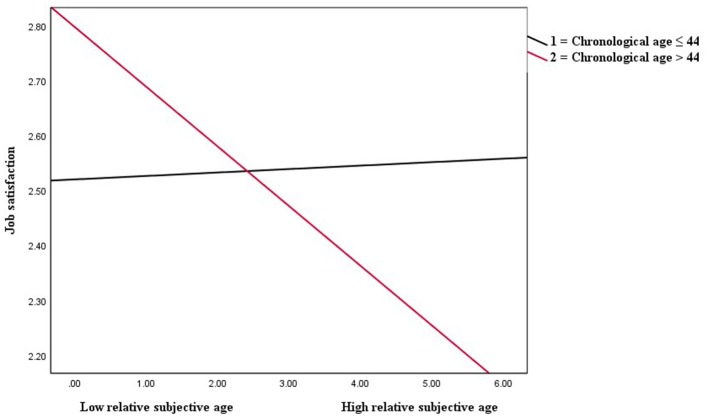
Chronological age as a moderator of the relationship between relative subjective age and job satisfaction.

The moderating effects of chronological age on the direct negative association of subjective age with job satisfaction (hypothesis 3), the positive relationship between subjective age and job burnout (hypothesis 4), and the indirect (via job burnout) negative subjective age-job satisfaction association (hypothesis 5) were examined using Hayes's PROCESS (model 8). The results presented in Table 4 demonstrated that the interaction term (relative subjective age × chronological age) had a significant negative effect on the direct negative association between subjective age and job satisfaction (*B* = −0.12, *p* < 0.05), showing that chronological age moderated the direct negative association of relative subjective age with job satisfaction. The interactions plotted at different values (chronological age ≤44 and chronological age > 44) of the moderator are shown in Figure 3. The strength of the negative subjective age-job satisfaction association was assessed using a simple slope test. The simple slope test showed that the negative subjective age-job satisfaction relationship was strong (*B* = −0.11, *p* < 0.01) for the respondents with chronological age >44 than for those with chronological age ≤44 (*B* = 0.01, ns). Thus, hypothesis 3 was supported.

**Table 4:** Moderated mediation analysis: Chronological age moderates the direct and indirect relationship between relative SA and job satisfaction (PROCESS model 8, 95% CI).

	**Job burnout**					**Job satisfaction**				
	**B**	**SE**	**T**	**LL**	**UL**	**B**	**SE**	**t**	**LL**	**UL**
Gender	−0.08	0.09	−0.85	−0.28	0.11	0.01	0.01	0.49	−0.02	0.03
Experience	0.01	0.01	0.05	−0.02	0.02	−0.04	0.12	−0.34	−0.28	0.20
Industry type	−0.01	0.01	−1.07	−0.03	0.01	−0.01	0.01	−0.42	−0.04	0.03
RSA	−0.03	0.07	−0.47	−0.18	0.11	0.48	0.19	2.56	0.11	0.86
Job burnout						0.27	0.06	4.20	0.14	0.39
Age	−0.52	0.19	−2.71	−0.89	−0.14	0.17	0.23	0.74	−0.28	0.62
RSA × Age	0.14	0.05	2.90	0.04	0.23	−0.12	0.04	−2.58	−0.20	−0.03
R^2^	0.15					0.10				
**Conditional direct effect of (actual) age**										
Age ≤ 44	0.10	0.03	3.13	0.04	0.16	0.01	0.03	0.20	−0.05	0.06
Age > 44	0.24	0.03	7.06	0.17	0.31	−0.11	0.03	−3.22	−0.18	−0.04
**Conditional indirect effect of chronological age**										
Age ≤ 44						−0.02	0.01		−0.04	−0.003
Age > 44						−0.04	0.02		−0.08	−0.01
**Index of moderated mediation**						**Index**	**Boot SE**		**LL**	**UL**
						−0.025		0.01	−0.06	−0.002

*n, 355; B, Unstandardized regression coefficient; SE, Standard error; Bootstrap sample size, 5,000; Confidence interval, 95%; LL, Lower limit; UL, Upper limit; RSA, Relative subjective age; Age, Chronological age*.

**Figure 4:**
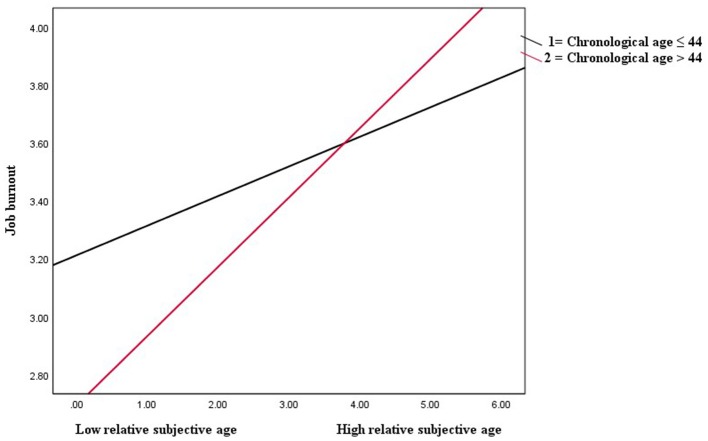
Chronological age as a moderator of the relationship between relative subjective age and job burnout.

Furthermore, the results presented in Table 4 depict that the interaction term (relative subjective age × chronological age) had a significant positive effect on the direct positive relationship between subjective age and job burnout (*B* = 0.14, *p* < 0.01), showing that chronological age moderated the direct positive association between relative subjective age and job burnout. The interactions plotted at different values (chronological age ≤44 years and chronological age > 44 years) of the moderator are shown in Figure 4. The strength of the positive association between relative subjective age and job burnout was assessed using the simple slope test. The simple slope test showed that the positive association between relative subjective age and job burnout was strong (*B* = 0.24, *p* < 0.001) for the respondents with chronological age > 44 years than for those with chronological age ≤44 years (*B* = 0.10, *p* < 0.05). Thus, the results supported hypothesis 4.

Finally, the results (Table 4) revealed that chronological age moderated the indirect association (via job burnout) between subjective age and job satisfaction [bootstrap estimate = −0.025, bias-corrected confidence interval (−0.06, −0.002)]. The results presented in Table 4 show that for employees with age >44 years, the indirect negative relationship between subjective age and job satisfaction was significant, while the indirect negative relationship between subjective age and job satisfaction was non-significant for employees with age ≤44 years. Thus, hypothesis 5 was also supported.

## Discussion

The present work, first, theorized and examined the relationship between subjective age and job satisfaction and, then, tested the mediating role of job burnout in this subjective age-job satisfaction relationship. Additionally, the study hypothesized that chronological age moderates both direct and indirect relationships between subjective age and job satisfaction. Time-lagged survey data collected from 355 employees in 62 service and manufacturing firms operating in Pakistan and analyzed using SEM and Hayes' PROCESS macro for SPSS supported the hypothesized relationships.

The results showed a significant negative relationship between subjective age and job satisfaction. Our findings suggest that employees with older subjective age bias may lack important resources, such as passion, energy, and skills that lead to a low level of job satisfaction. Our findings concord with previous empirical studies on subjective age in the work context that suggest that subjective is negatively related to employees' work-related outcomes (23, 43, 52), and life satisfaction (44).

Our study revealed that job burnout mediates the negative relationship between subjective age and job satisfaction, indicating that older subjective age bias reflects limited FTP and results in employees' personal resources' depletion, and continual resources' depletion can invoke employees' feelings of job burnout. Thus, our findings also extend support to existing studies [e.g., (47, 48)] that posit that subjective age predicts the depletion of individuals' resources, such as physical and mental health and energy, which symptomizes as anxiety, helplessness, depression and decreased self-esteem feelings (34, 51). Seen in this way, our findings concord with the COR theory (26), which suggests that employees' lack of personal resources, such as emotional and physical energy, can result in job burnout and negatively influences their work-related positive outcomes, such as job satisfaction and job performance.

Finally, the present study showed that as compared to older subjective age bias among younger employees, older subjective age bias among middle-aged employees has a more profound effect on job burnout and job satisfaction. Given the importance of subjective age for theory and practice and the lack of research on the subjective age-job satisfaction relationship, the present study is timely and relevant. Importantly, the present work is based on a diverse sample that belonged to 62 firms operating in different manufacturing and service sectors. Data from the diverse sample is one of the key highlights of the present study, as such a diverse sample allowed us to capture maximum variance and also enhanced the generalizability (55).

### Theoretical Contributions

This study makes several contributions to theory by theorizing the subjective age-job satisfaction relationship. Although there is abundant literature on the age-job satisfaction connection (8, 12, 68), the analysis of the literature revealed that the existing literature did not pay attention to the notion of subjective age in the age-job satisfaction link. We attempted to go some way to fill in this void and, importantly, we provided empirical support to the literature [e.g., (20, 21)] that suggests that subjective age can better explain the variations and complexities in employees' work-related outcomes and capture the diversity of the dimensions of the age-job satisfaction relationship. As a key contribution to the literature on subjective age (15, 24), the study established job burnout as a mediator of the subjective age-job satisfaction association. Furthermore, by explaining these relationships using the SST and the COR perspectives, the work at hand responded to the calls for theory-driven research on age and employees' work-related outcomes (22, 23) and advanced the scope of the SST and the COR theory.

Finally, by establishing chronological age as a boundary condition of the subjective age-job satisfaction link, the present study not only explored the complexities involved in this subjective age-job satisfaction connection and enhanced our understanding of when subjective age (24, 51) can have a more pronounced effect on job burnout and job satisfaction but also contributed to the job burnout literature (42, 45, 46) by extending the nomological networks of its antecedents and outcomes. In essence, our study not only theorized and empirically showed that subjective age is negatively related to job satisfaction but also brought to the fore the complex nature of this relationship by establishing job burnout as an important underlying mechanism of the subjective age-job satisfaction association and foregrounding chronological age as a boundary condition of both direct and indirect associations between subjective age and job satisfaction.

### Practical Implications

Subjective age higher than the chronological age can have important practical implications for job satisfaction that can influence employees' productivity and performance (43, 52, 69, 70). Our findings suggest that by broadening the measurement and meaning of age, managers can understand an alternative explanation of age and its consequences for employees' work-related outcomes. Consequently, managers can develop policies and strategies to triumph over the factors that can increase employees' subjective age, job burnout, and a low level of job satisfaction. For instance, past research suggests that a sensed lack of control over one's work can lead to older age subjective bias (15, 16, 20). Managers can empower employees to counter such feelings and reduce their older subjective bias.

Moreover, although the aim of this study was not to foreground the role of subjective age in countering age stereotyping, following (71, 72), we suggest that understanding employees' subjective age can help managers counter the age stereotyping at the workplace. The focus on subjective age also provides some critical insights into the aging population and workforce (73) because of the likely difference between chronological age and subjective age and the implications of this difference for employees' behaviors, preferences, and actions. Thus, we suggest that the concepts of age and aging in organizations need to be revisited to make these concepts more relevant and meaningful.

### Limitations and Future Research

This study is not without limitations. Reliance on self-reported measures presents a key limitation and should be taken care of when explaining and making sense of the findings of this study. Moreover, although the findings of present work are based on time-lagged data, our research design restricts drawing causal inferences. Future studies can use longitudinal and experimental designs to establish causality. Likewise, our findings reflect that employees' perceptions of subjective age and, thus, do not inform us about managers' perceptions of subjective age and its implications for both managers' and employees' work-related outcomes. Future studies can collect data from managers to understand the managerial perspective of subjective age to offer further theoretical and practical insights.

Additionally, subjective age negatively affects psychological well-being and mental and physical health (15, 16) that, in turn, can negatively affect job satisfaction, suggesting that factors, such as psychological well-being and mental and physical health, can mediate the subjective age-job satisfaction link. Likewise, different individual-level differences, such as employees' perception of meaningful work can moderate the subjective age-job satisfaction relationship, as employees who perceive that their work is valuable and contributes to greater good motivations (61) can be motivated to enhance new skills and knowledge. Thus, they may not perceive the deficiency of resources to perform their work roles and feel less burned out despite older subjective age bias. Given the paucity of research on the mediating and moderating variables of the subjective age-job satisfaction link, it is important to examine the intervening factors and the boundary conditions of the subjective age-job satisfaction association to foreground the intricate nature of this relationship.

Finally, this study is contextualized in Pakistan a country where collectivist culture prevails (74, 75). Future studies in different contexts, industries and cultures can further enhance our understanding of the subjective age-job satisfaction link. The implications of subjective age for age stereotyping offer an important area of future research. The relationship of subjective age with employees' performance and productivity is also an important theme that needs to be investigated.

## Conclusion

Building on the SST and the COR theory, the present study hypothesized and empirically tested the direct and indirect (via job burnout) relationship between subjective age and job satisfaction. Moreover, the work at hand hypothesized and empirically tested chronological age as a moderator of the direct and indirect relationships of subjective age with job satisfaction. By collecting survey data in three waves (2 months apart) from 355 employees in 62 firms operating in various service and manufacturing industry sectors in Pakistan and analyzing data using structural equation modeling, PROCESS macro for SPSS, and bootstrapping technique, the present study showed that subjective age is negatively related to job satisfaction, both directly and indirectly, via job burnout. By doing so, the present work provided important alternative vantage points to explore the age-job satisfaction association and responded to the calls for theory-driven research on the age-job satisfaction connection (22, 23). Finally, by establishing chronological age as a boundary condition of the subjective age-job satisfaction association and the subjective age-job burnout connection, the present study foregrounded the complexities involved in these relationships and enhanced our understanding of when subjective age has a more pronounced effect on job burnout and job satisfaction. Our findings suggest that managers can comprehend negative implications of age for employees' work-related outcomes more effectively by broadening the measurement and meaning of age.

## Data Availability Statement

The datasets generated for this study are available on request to the corresponding author.

## Ethics Statement

This study involving human participants was reviewed and approved by the Ethics Committee of the Department of Management Sciences, COMSATS University Islamabad, Lahore Campus, Lahore, Pakistan. The participants provided their written informed consent to participate in this study.

## Author Contributions

MA, MKA, MU, and FA: definition of research objectives, models, hypotheses, data analysis plan, principal article writing, article revision and proofreading, and final approval. MA, FA, and MU: the provision of materials (i.e., questionnaires) and data collection. MKA, MA, FA, and MU: data analysis.

### Conflict of Interest

The authors declare that the research was conducted in the absence of any commercial or financial relationships that could be construed as a potential conflict of interest.
